# Intraoperative cerebral desaturation during low-central-venous-pressure hepatectomy with intermittent Pringle maneuver: a case report

**DOI:** 10.3389/fmed.2026.1880040

**Published:** 2026-06-22

**Authors:** Yan Wei, Yanqin Mi, Zhengfang Qi, Yatao Liu, Zhaohui Gao

**Affiliations:** 1 The First School of Clinical Medicine of Lanzhou University, Lanzhou, China; 2 Department of Anesthesiology and Operation, First Hospital of Lanzhou University, Lanzhou, China

**Keywords:** cerebral desaturation, hepatectomy, low central venous pressure, near-infrared spectroscopy, postoperative delirium, Pringle maneuver

## Abstract

**Background:**

Intraoperative cerebral desaturation has been associated with postoperative neurocognitive complications and may represent a potentially modifiable perioperative risk factor. The Pringle maneuver, or intermittent portal triad clamping, is routinely used to minimize blood loss during low-central-venous-pressure (LCVP) hepatectomy; however, its real-time relationship with cerebral oxygenation remains insufficiently characterized. We report a case providing high-resolution, time-locked documentation of abrupt bilateral regional cerebral oxygen saturation (rSO₂) decline during repeated intermittent hepatic inflow occlusion under LCVP conditions, followed by transient CAM-positive postoperative delirium.

**Case presentation:**

A 51-year-old female underwent elective right hepatectomy for hepatocellular carcinoma under low-central-venous-pressure (CVP < 5 cm H₂O) anesthesia. Bilateral rSO₂ was continuously monitored using near-infrared spectroscopy (NIRS) during eight cycles of intermittent Pringle maneuver (10–15 min occlusion/5 min reperfusion). rSO₂ showed a progressive downward trend across repeated clamping cycles, reaching a nadir of 33% on the left and 30% on the right 6 min into the eighth occlusion, coinciding with a CVP decrease to 0.9 cm H₂O despite MAP remaining above 65 mmHg. The patient developed transient CAM-positive, predominantly hypoactive postoperative delirium from POD 1 to POD 3, documented during the morning assessments on POD 1 and POD 2 and during the evening assessment on POD 3. The delirium resolved by POD 4 without pharmacological treatment. Structured follow-up at 1 month revealed subtle impairments in executive function and episodic memory (MoCA-Basic 25/30), which normalized to baseline (28/30) at the 3-month assessment. Evaluations at 6 months and 1 year demonstrated sustained neurocognitive recovery with no functional deficits or oncological recurrence.

**Conclusion:**

This case suggests that intermittent portal triad clamping under LCVP conditions may be associated with clinically important cerebral desaturation, even when conventional mean arterial pressure targets appear acceptable. The observation highlights the limitation of relying solely on systemic arterial pressure to infer cerebral oxygenation during high-risk hepatectomy. NIRS may serve as a useful adjunctive monitor for detecting otherwise unrecognized cerebral desaturation and may help guide timely multidisciplinary responses. However, the relationship between intraoperative cerebral desaturation and postoperative delirium in this case should be interpreted as hypothesis-generating rather than causal.

## Introduction

Hepatectomy remains a cornerstone therapy for liver malignancies (1). Advances in surgical technique and perioperative care have reduced perioperative mortality in contemporary series, although major liver resection continues to impose substantial physiological stress (2). To minimize intraoperative blood loss and improve surgical exposure, low-central-venous-pressure (LCVP) anesthesia is commonly combined with intermittent portal triad clamping, also known as the Pringle maneuver (3, 4). While these strategies are effective for surgical hemostasis, they may also alter venous return, preload reserve, and systemic hemodynamics, particularly during repeated clamping-reperfusion cycles. Their potential real-time relationship with cerebral oxygenation during hepatectomy remains insufficiently characterized.

Postoperative delirium (POD) is a common neuropsychiatric complication after major abdominal surgery and is associated with adverse short- and long-term outcomes. Near-infrared spectroscopy (NIRS) allows continuous, noninvasive monitoring of regional cerebral oxygen saturation (rSO₂) and has been investigated as a tool for detecting intraoperative cerebral desaturation in several surgical settings. However, its application in liver surgery remains relatively limited. A prospective observational study by Collin et al. (5) reported frequent but generally modest rSO₂ declines during liver surgery, but the temporal relationship between specific intra-abdominal maneuvers and abrupt cerebral desaturation has not been fully defined.

Here, we report a case of right hepatectomy under LCVP anesthesia in which continuous NIRS captured a severe, time-locked bilateral rSO₂ decline during the eighth cycle of intermittent Pringle maneuver, despite apparently acceptable mean arterial pressure. The patient subsequently developed transient CAM-positive hypoactive POD. By presenting the intraoperative physiological trends and postoperative course, this report aims to highlight the potential dissociation between systemic arterial pressure and cerebral oxygenation during high-risk hepatectomy, and to generate a cautious hypothesis regarding the possible contribution of repeated inflow occlusion under reduced preload reserve to cerebral desaturation and postoperative neurocognitive vulnerability.

## Case presentation

A 51-year-old woman (BMI 22.5 kg/m^2^) was admitted for an elective right hepatectomy following a multidisciplinary tumor board review. Four months prior, she was diagnosed with multifocal hepatocellular carcinoma (HCC) secondary to chronic hepatitis B. Initial magnetic resonance imaging (MRI) revealed multiple enhancing hepatic lesions accompanied by retroperitoneal lymphadenopathy and limited ascites.

She underwent two sessions of transarterial chemoembolization and sequential systemic therapy, after which imaging demonstrated a partial response with the residual dominant tumor confined to the right liver lobe, allowing curative resection.

Her past medical history was notable for well-controlled essential hypertension (indapamide 2.5 mg daily) and a benign left-breast mass excision in 2018. She had no history of tobacco or alcohol use, and no prior blood transfusions. Her family history was positive for hepatitis B but negative for malignancy. She reported allergies to amoxicillin and penicillin.

### Pre-operative assessment

Physical examination and vital signs were within normal limits. Preoperative laboratory investigations revealed preserved hepatic and renal function (bilirubin 10.4 μmol/L, albumin 38 g/L, creatinine 44.9 μmol/L, INR 1.12) with a hemoglobin level of 133 g/L and a platelet count of 72 × 10^9^/L. The patient was categorized as Child–Pugh class A. Notably, her baseline neurocognitive status was intact, as evidenced by a Montreal Cognitive Assessment-Basic (MoCA-Basic) Chinese Version score of 28/30.

### Anaesthetic management

Standard monitoring was supplemented with right radial arterial and internal jugular venous catheters for continuous hemodynamic tracking. A low-central-venous-pressure (CVP < 5 cm H₂O) strategy was achieved using restrictive crystalloid administration combined with low-dose norepinephrine. Bilateral frontal near-infrared spectroscopy (NIRS; INVOS, Mindray) was applied to continuously monitor regional cerebral oxygen saturation (rSO₂). Baseline rSO₂ was defined as the stable bilateral value recorded after induction and before the first Pringle maneuver under stable hemodynamic and ventilatory conditions. The baseline rSO₂ values were 66% on the left and 61% on the right. In our institutional practice, clinically relevant cerebral desaturation prompted intervention when rSO₂ decreased by more than 20% from baseline or fell below an absolute value of 50%. General anesthesia was induced with dexamethasone, penehyclidine, ciprofol, sufentanil, and rocuronium, and maintained via continuous intravenous infusion of ciprofol, remifentanil, and dexmedetomidine. After tracheal intubation, mechanical ventilation was delivered using volume-controlled ventilation. Before the cerebral desaturation episode, FiO₂ was maintained at 0.40, tidal volume was set at 6–8 mL/kg, PEEP was set at 0 cmH₂O to facilitate the low-central-venous-pressure strategy, respiratory rate was 12 breaths/min, and the inspiratory-to-expiratory ratio was 1:2. Airway pressure was maintained below 30 cmH₂O. Ventilation was adjusted to maintain normocapnia, with EtCO₂ generally kept within 35–45 mmHg. No deliberate hypocapnia strategy was used.

### Intra-operative course

**Figure 1:**
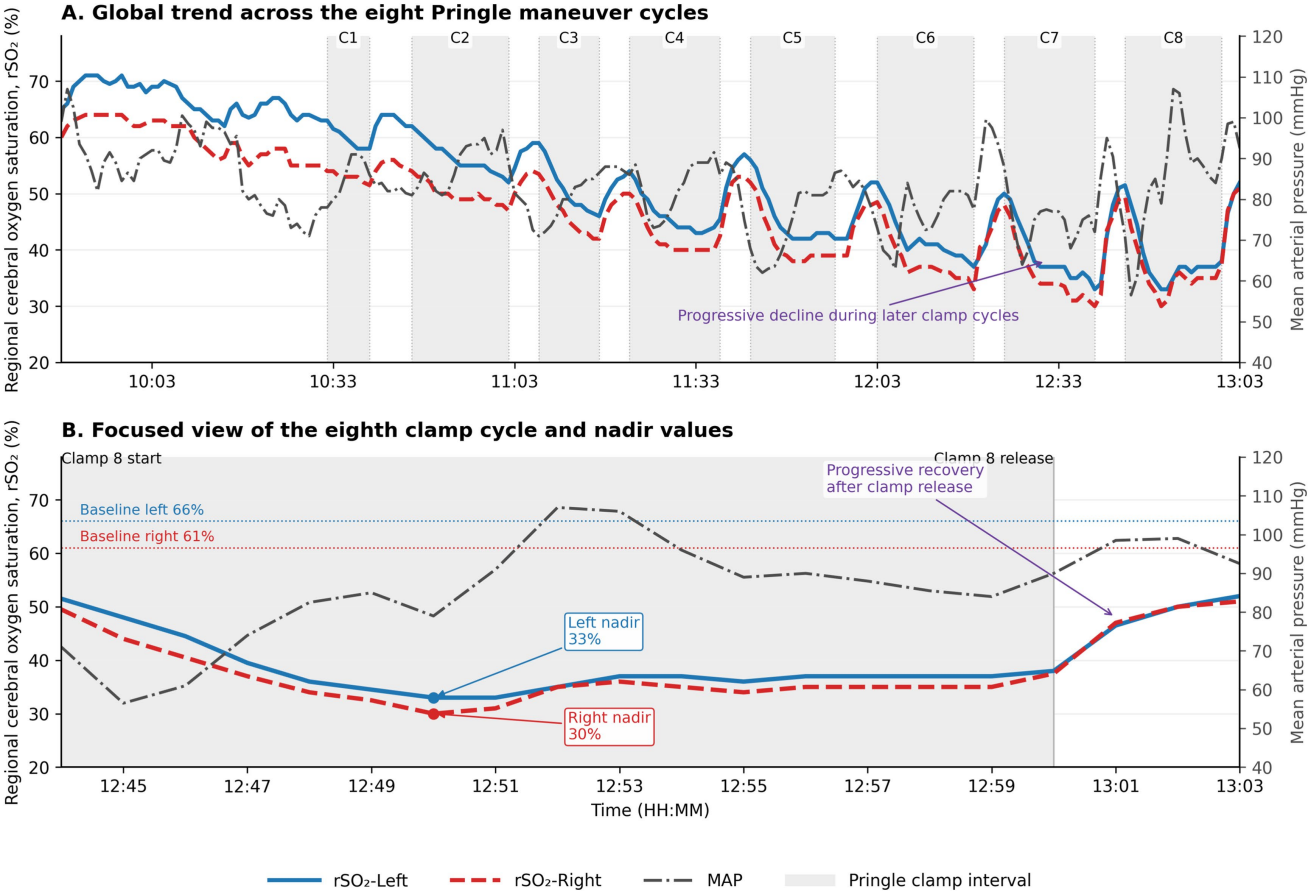
Intraoperative cerebral oxygenation and hemodynamic trends during eight Pringle maneuver cycles. **(A)** Global trend of bilateral rSO₂ and MAP throughout eight Pringle maneuver cycles. The solid blue line indicates left rSO₂, the dashed orange line indicates right rSO₂, and the gray dash-dot line indicates MAP on the right y-axis. Shaded bands represent Pringle clamp intervals labeled C1–C8. **(B)** Focused view of the eighth clamp cycle. Baseline rSO₂ values were 66% on the left and 61% on the right. During the eighth clamp, rSO₂ decreased to 33 and 30%, corresponding to approximately 50 and 51% reductions from baseline, respectively. Clamp start and release are marked, and early reperfusion was followed by progressive rSO₂ recovery. All axes, units, time points, and clamp intervals are clearly labeled and remain interpretable in greyscale.

Following abdominal exploration, a curative right hepatectomy proceeded. Parenchymal transection was facilitated by eight cycles of intermittent Pringle maneuver (10–15 min clamp, 5 min reperfusion) under strict LCVP management (CVP < 5 cm H₂O). Time-resolved hemodynamic and cerebral oxygenation data across the eight Pringle maneuver cycles are summarized in Table 1. Across repeated clamping cycles, rSO₂ showed a progressive downward trend, with clinically relevant decreases becoming more apparent during later cycles (Table 1, Figure 1A). During the eighth clamp, while a major vein was being divided, rSO₂ reached a nadir of approximately 33% on the left and 30% on the right 6 min into the occlusion, corresponding to relative reductions of approximately 50 and 51% from baseline, respectively (Figure 1B). This episode coincided with a CVP decrease to 0.9 cm H₂O, despite MAP around the rSO₂ nadir remaining above the conventional threshold of 65 mmHg. Because premature reperfusion would jeopardize hemostasis during the division of a major vein, the surgeon maintained the clamp for the full 15-min interval. The anesthesia team responded by titrating norepinephrine to 0.04 μg/kg/min and increasing FiO₂ from 0.40 to 0.60. In addition, hypocapnia was avoided, and ventilation was adjusted as appropriate to maintain EtCO₂ toward the high-normal range, with a target of approximately 40–45 mmHg, to support cerebral blood flow. At the rSO₂ nadir, EtCO₂ was approximately 36 mmHg. Other ventilatory settings, including tidal volume and PEEP, were not aggressively increased because systemic oxygenation was preserved and further increases in airway pressure or PEEP could have compromised venous return under LCVP conditions.

**Table 1:** Time-resolved hemodynamic and cerebral oxygenation data across the eight Pringle maneuver cycles.

Cycle	Clamp interval	Duration (min)	HR, bpm median (range)	MAP, mmHg median (range)	CVP, cmH₂O median (range)	Lowest rSO₂ L/R (%)	Interpretation
Baseline	09:48	—	70	99	4.2	66/61	Reference before first clamp
C1	10:32–10:39	7	70 (66–75)	86 (77–94)	3.3 (2.8–4.3)	58/52	Mild decline
C2	10:46–11:02	16	71 (67–74)	88 (80–100)	3.3 (0.5–4.7)	52/47	Mild-to-moderate decline
C3	11:07–11:17	10	72 (69–76)	80 (69–85)	2.7 (1.9–3.5)	46/42	Progressive decline
C4	11:22–11:37	15	71 (63–74)	84 (70–94)	1.8 (1.4–3.1)	43/39	Progressive decline
C5	11:42–11:56	14	74 (71–76)	72 (61–83)	0.4 (0.0–1.6)	41/38	Low CVP with further rSO₂ decline
C6	12:03–12:19	16	78 (70–85)	76 (63–85)	1.4 (0.1–1.9)	38/34	Later-cycle decline
C7	12:24–12:39	15	81 (69–85)	75 (61–124)	1.5 (0.7–3.3)	35/32	Clinically relevant desaturation
C8	12:44–13:00	16	82 (71–89)	85 (53–107)	1.2 (0.7–3.4)	33/30	Severe bilateral desaturation during major vein division

HR, MAP, and CVP are reported as median (range) within each recorded clamp interval. rSO₂ values are shown as left/right nadirs and rounded to the clinically annotated trend values shown in Figure 1. Ventilation remained volume-controlled with tidal volume 6–8 mL/kg, PEEP 0 cmH₂O, peak airway pressure <30 cmH₂O, and EtCO₂ generally maintained within 35–45 mmHg. FiO₂ was 0.40 before the event and increased to 0.60 during rescue in C8. CVP, central venous pressure; HR, heart rate; MAP, mean arterial pressure; rSO₂, regional cerebral oxygen saturation. The shaded row indicates the eighth clamp cycle during which severe bilateral cerebral desaturation occurred.

Arterial blood gas analysis at the rSO₂ nadir showed mild metabolic acidosis (pH 7.29, lactate 2.3 mmol/L), while systemic oxygenation (PaO₂ 141 mmHg) and hemoglobin concentration (12.1 g/dL) were preserved. These findings made severe hypoxemia or anemia unlikely to be the primary driver of the abrupt rSO₂ decline, but the underlying mechanism cannot be confirmed from blood gas data alone. Total estimated blood loss was ~500 mL, managed with 600 mL of packed red blood cells and 400 mL of fresh frozen plasma (Table 2).

**Table 2:** Intra-operative arterial blood gas results.

Parameter (reference range)	Pre-Clamp	rSO₂ nadir	Post-Clamp
pH (7.35–7.45)	7.36	7.29	7.34
PCO₂ (mmHg, 35–45)	42	43	45
PO₂ (mmHg, ≥80)	146	141	87
Na^+^ (mmol/L, 135–145)	138	135	137
K^+^ (mmol/L, 3.5–5.3)	4.0	5.8	5.6
Ca^2+^ (mmol/L, 1.12–1.32)	1.34	1.27	1.21
Glu (mmol/L, 4–10)	8.0	14.2	8.8
Lac (mmol/L, <2)	0.8	2.3	1.9
Hct (%, 42–49)	39	35	36
HCO_3_^−^ (mmol/L, 22–26)	23.7	20.5	24.3
BE (mEq/L, −3 to +3)	−1.8	−5.9	−1.5
SO₂ (%, 95–100)	98.7	98.5	96.8
tHb (g/dL, 12–16)	12.8	12.1	12.7

Reference ranges are based on institutional laboratory standards. Highlighted values indicate clinically relevant deviations and should be interpreted in the perioperative context.

### Postoperative outcome

Following uneventful extubation in the post-anesthesia care unit, the patient was transferred directly to the surgical ward. Postoperative biochemistry demonstrated a transient ischemia–reperfusion response, with aspartate aminotransferase (225 U/L), alanine aminotransferase (95 U/L), and C-reactive protein (31.6 mg/L) peaking between POD 1 and POD 4.

### Postoperative delirium assessment

Postoperative delirium was assessed using the confusion assessment method (CAM), a validated delirium screening tool, by an anesthesiologist specifically responsible for postoperative follow-up and trained in CAM administration (6, 7). Assessments were performed twice daily, in the morning and evening, for 7 postoperative days or until hospital discharge. CAM-positive hypoactive delirium was documented from POD 1 to POD 3, specifically during the morning assessments on POD 1 and POD 2 and during the evening assessment on POD 3. The episodes were characterized by acute and fluctuating inattention, temporal and spatial disorientation, intermittent hallucinations, and inability to correctly answer simple CAM assessment questions. No formal delirium severity scale was applied. Because delirium was assessed at scheduled twice-daily intervals rather than by continuous observation, the exact duration of each individual episode could not be precisely determined.

The delirium resolved by POD 4 without pharmacological treatment in the ward. Intraoperative dexmedetomidine exposure is described above as part of anesthetic maintenance; however, no postoperative sedative or antipsychotic medication was administered for delirium. The surgical team evaluated potential alternative explanations, including hepatic encephalopathy and hyperammonemia, which were not supported. During the delirium period, there was no evidence of hypoxemia, infection, hypoglycemia, major electrolyte disturbance, uncontrolled pain, or opioid overdose.

Structured neurocognitive screening was performed using the Chinese version of the Montreal Cognitive Assessment-Basic (MoCA-Basic/MoCA-BC), according to our institutional perioperative follow-up protocol. Our center serves a population in Northwest China with heterogeneous educational backgrounds, and many middle-aged and older surgical patients have limited formal education. Therefore, MoCA-Basic has been adopted in our center as a standardized cognitive screening tool for perioperative follow-up studies. To avoid confusion with alternate forms of the standard MoCA, we have spelled out “MoCA-Basic” throughout the revised manuscript rather than using the abbreviation “MoCA-B.” Assessments were performed by the same trained examiner at baseline, 1 month, and 3 months. The baseline MoCA-Basic score was 28/30. At 1 month, the patient showed mild cognitive fluctuation, mainly involving executive function and episodic memory, with a score of 25/30. The score returned to baseline at 3 months (28/30). These longitudinal results were interpreted as descriptive cognitive screening follow-up rather than definitive evidence of perioperative neurocognitive disorder (28).

## Discussion

This case report documents a profound and precipitous bilateral cerebral desaturation (rSO₂ ≈ 30%) during the eighth cycle of an intermittent Pringle maneuver in a patient undergoing low-central-venous-pressure (LCVP) hepatectomy. The clinical significance of this episode was supported by both absolute and relative criteria: rSO₂ fell below 50% bilaterally and decreased by approximately 50% from baseline on both sides, far exceeding our institutional intervention criterion of a > 20% relative decline or an absolute rSO₂ value below 50%. Notably, this severe desaturation episode occurred despite the maintenance of conventional hemodynamic targets, with MAP around the rSO₂ nadir remaining above the conventional threshold of 65 mmHg.

Current Enhanced Recovery After Surgery (ERAS) guidelines for hepatic resection recommend LCVP strategies to minimize intraoperative blood loss and improve surgical visibility (8, 9). The Pringle maneuver remains a widely used technique for controlling hepatic inflow (10, 11). However, when these strategies are combined, they may alter venous return, preload reserve, and systemic hemodynamics (3, 4). Recent prospective observational studies have shown that rSO₂ declines may occur during liver surgery (5, 12), but the temporal relationship between specific surgical maneuvers and abrupt cerebral desaturation remains incompletely characterized. This case provides high-resolution, time-locked documentation that repeated inflow occlusion under LCVP conditions may be associated with clinically important cerebral desaturation, even when systemic arterial pressure appears acceptable. This observation should not be interpreted as contradicting ERAS recommendations or conventional MAP targets, which remain important population-based principles for perioperative hemodynamic management. Rather, this case suggests that selected high-risk patients may have individual vulnerability in which MAP alone does not fully reflect cerebral oxygenation, and that NIRS may provide complementary information.

Ventilatory management was also considered when interpreting this cerebral desaturation event. Hypocapnia may reduce cerebral blood flow through cerebral vasoconstriction, whereas maintaining end-tidal carbon dioxide within the normal-to-high-normal range may help preserve cerebral perfusion. In this case, the patient was ventilated with volume-controlled ventilation, FiO₂ was 0.40 before the event and was increased to 0.60 during rescue, tidal volume was maintained at 6–8 mL/kg, PEEP was set at 0 cmH₂O to facilitate the LCVP strategy, airway pressure was kept below 30 cmH₂O, and EtCO₂ was maintained within the normocapnic range. During the rescue phase, hypocapnia was avoided and ventilation was adjusted as appropriate toward a high-normal EtCO₂ target of approximately 40–45 mmHg to support cerebral blood flow. Aggressive increases in PEEP, tidal volume, or recruitment pressure were not pursued because systemic oxygenation was preserved and such maneuvers could have further impaired venous return under LCVP conditions. These findings make severe systemic hypoxemia or carbon dioxide-related cerebrovascular changes less likely to be the primary explanation for the abrupt rSO₂ decline, although ventilatory factors cannot be completely excluded as contributing factors.

### Possible hemodynamic explanation: reserve depletion during repeated Pringle maneuvers under LCVP

The abrupt cerebral desaturation observed during the eighth Pringle maneuver may plausibly reflect an interaction between strict LCVP management and the hemodynamic effects of repeated portal triad clamping. LCVP is commonly used during hepatic resection to reduce hepatic venous bleeding, whereas the Pringle maneuver transiently alters venous return and systemic hemodynamics (13, 14). In this case, MAP was maintained above 65 mmHg with vasopressor support, but this did not necessarily ensure preserved cardiac output or adequate cerebral oxygen delivery. The very low CVP observed during the desaturation episode (0.9 cmH₂O) is consistent with reduced preload reserve. Under these conditions, repeated clamping may have progressively narrowed the patient’s compensatory margin, making cerebral oxygenation vulnerable despite apparently acceptable systemic arterial pressure (15, 16).

A notable feature of this case is that severe cerebral desaturation occurred during the eighth Pringle maneuver cycle rather than during the preceding cycles. This pattern may represent a cumulative reserve-depletion or threshold phenomenon rather than the effect of a single clamp episode alone. Repeated clamping-reperfusion cycles, restrictive fluid administration, sustained LCVP, vasopressor-supported MAP, and the late phase of parenchymal transection may have collectively reduced the patient’s hemodynamic reserve. If compensatory reserve had become limited, the eighth clamp may have been sufficient to precipitate a sharp fall in rSO₂ within the standard 15-min occlusion window (17, 18). Importantly, this interpretation remains hypothesis-generating because cardiac output, jugular venous pressure, cerebral blood flow, and neuroimaging evidence of cerebral ischemia were not directly measured.

### Hypothesis-generating interpretation: a possible “two-hit” model

Postoperative delirium (POD) is a prevalent neuropsychiatric complication following major abdominal surgery and is associated with prolonged hospitalization and long-term cognitive decline (19, 20). In the present case, POD should be interpreted as a multifactorial outcome rather than being attributed solely to the intraoperative desaturation event. Potential contributing factors included major oncologic liver surgery, hepatic ischemia–reperfusion response, systemic inflammation, perioperative transfusion, metabolic stress, anesthetic exposure, and the general physiological burden of major abdominal surgery.

**Figure 2:**
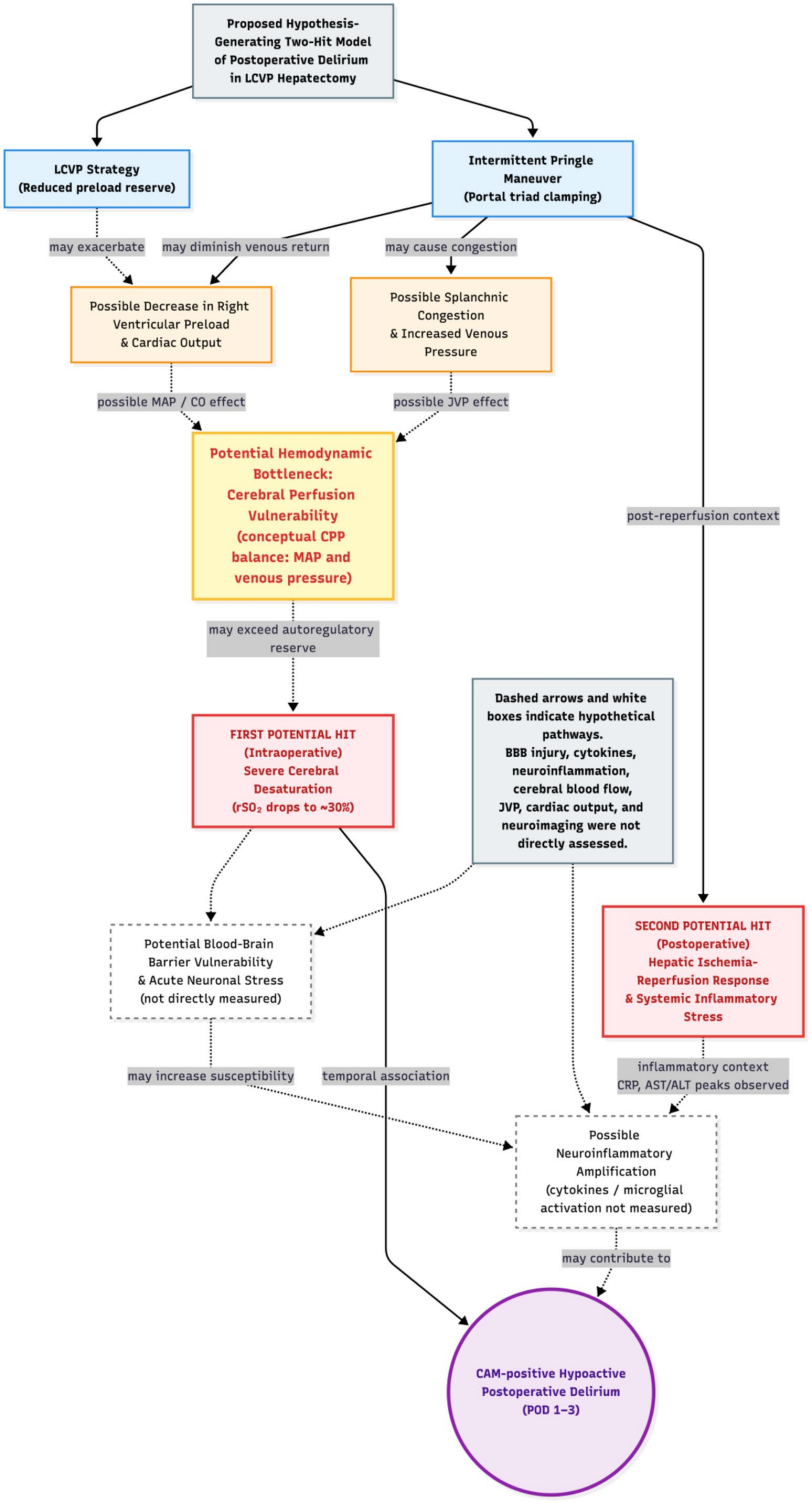
Proposed hypothesis-generating “two-hit” model of postoperative delirium in LCVP hepatectomy with intermittent Pringle maneuver. This conceptual diagram illustrates a hypothetical pathway linking low-central-venous-pressure anesthesia, intermittent portal triad clamping, severe intraoperative cerebral desaturation, postoperative hepatic ischemia–reperfusion response, and CAM-positive hypoactive postoperative delirium. The first potential hit is severe intraoperative cerebral desaturation during the eighth Pringle maneuver, possibly reflecting cerebral oxygen delivery mismatch under reduced preload reserve. The second potential hit is postoperative systemic inflammatory stress after hepatic ischemia–reperfusion. This model is hypothesis-generating only. Blood–brain barrier injury, cytokine activation, neuroinflammation, cerebral blood flow, jugular venous pressure, cardiac output, and neuroimaging findings were not directly assessed in this case; therefore, the pathway should not be interpreted as an established causal mechanism.

Within this multifactorial context, the temporal association between severe intraoperative cerebral desaturation and subsequent transient CAM-positive hypoactive POD raises the possibility of a “two-hit” process; however, this interpretation remains hypothetical in a single case (Figure 2). The first potential component was severe intraoperative cerebral desaturation (rSO₂ approximately 30%), which may have reflected cerebral oxygen delivery mismatch and could have increased neurocognitive vulnerability. Although blood–brain barrier (BBB) integrity was not directly assessed in this case, previous evidence suggests that perioperative cerebral stress may be associated with BBB vulnerability under certain conditions (21).

The second potential “hit” was proposed on the basis of the postoperative hepatic ischemia–reperfusion response and systemic inflammatory stress, reflected by transient elevations in AST/ALT and CRP. However, these laboratory changes should be interpreted only as markers of postoperative systemic stress and do not establish a direct temporal or causal link with the CAM-positive POD episodes (15, 22). Experimental and clinical literature suggests that peripheral inflammatory mediators may interact with a vulnerable brain through BBB-related pathways, microglial activation, and neuroinflammatory signaling, all of which have been implicated in POD pathogenesis (11, 23). In the present case, however, cytokine activation, BBB injury, microglial activation, neuroinflammatory biomarkers, and neuroimaging evidence of cerebral ischemia or edema were not assessed. Therefore, the proposed two-hit pathway should be interpreted as a hypothesis-generating framework rather than an established mechanism. Existing evidence suggests that NIRS-guided optimization may reduce POD in selected surgical populations by identifying and correcting cerebral desaturation, but these findings cannot be directly extrapolated to prove causality in this single hepatectomy case (12, 24–26). The longitudinal follow-up is compatible with this clinical trajectory, but it does not establish a causal relationship between the intraoperative desaturation event and postoperative neurocognitive symptoms.

### Clinical implications and monitoring strategies

This case does not challenge the importance of maintaining appropriate systemic hemodynamic targets. Instead, it highlights that an apparently acceptable MAP during LCVP hepatectomy may not always fully reflect cerebral oxygenation during repeated Pringle maneuver. In selected high-risk hepatectomy patients, NIRS may serve as an adjunctive monitor to detect otherwise unrecognized cerebral desaturation (5, 24). When severe rSO₂ decline occurs during portal triad clamping, management may include reassessment of oxygenation, hemoglobin concentration, carbon dioxide tension, vasoactive support, volume status, and the surgical clamping strategy (13, 16). If anesthetic optimization is insufficient and surgical conditions permit, temporary clamp release, shorter subsequent clamping intervals, or brief surgical pauses may be considered (10, 15). These implications remain hypothesis-generating and require validation in prospective studies.

## Limitations

Several limitations should be acknowledged. First, as a single case report, this study cannot establish causality between the Pringle maneuver, cerebral desaturation, and postoperative delirium. POD is multifactorial, and major oncologic liver surgery, hepatic ischemia–reperfusion response, systemic inflammation, transfusion, metabolic stress, anesthetic and vasoactive drug exposure, sleep disruption, and the overall burden of major abdominal surgery may all have contributed. Perioperative medications, including dexmedetomidine, remifentanil, ciprofol, norepinephrine, and dexamethasone, may have influenced cerebral hemodynamics or POD risk, although their individual contributions cannot be determined. Second, although POD was assessed twice daily using CAM by a trained anesthesiologist, no formal delirium severity scale was applied, episode duration was not continuously measured, and no independent neurological or psychiatric consultation was performed (27). Serial MoCA-Basic follow-up should be interpreted cautiously because it is a screening assessment rather than a comprehensive neuropsychological battery, and practice effects from repeated administration cannot be excluded. Third, NIRS reflects regional cerebral oxygen saturation rather than direct cerebral blood flow or ischemia, and rSO₂ values may be affected by sensor position, extracranial contamination, arterial carbon dioxide tension, hemoglobin concentration, and systemic hemodynamics. Finally, cardiac output, jugular venous pressure, cerebral blood flow, blood–brain barrier injury markers, inflammatory cytokines, neuroinflammatory biomarkers, and neuroimaging were not available. Therefore, the proposed “two-hit” model should be interpreted as hypothesis-generating rather than confirmatory.

## Conclusion

Severe cerebral desaturation may occur during repeated Pringle maneuver under LCVP anesthesia despite apparently adequate MAP. In this case, NIRS revealed a time-locked rSO₂ decline that was not reflected by conventional MAP monitoring alone. The subsequent CAM-positive hypoactive POD was likely multifactorial, and the proposed “two-hit” model should be viewed as hypothesis-generating. Prospective studies are needed to clarify whether NIRS-guided multidisciplinary interventions can improve neurocognitive outcomes after hepatectomy.

## Patient perspective

When I was told I needed liver surgery, my biggest fear was not just the cancer itself, but whether I would wake up ‘not feeling like myself’ or lose my memory. On the first few days after surgery, I did feel confused and had trouble focusing, which was very frightening for my family. However, the doctors explained that they had closely monitored my brain oxygen levels during the operation and adjusted my medications promptly to protect my brain. I am incredibly relieved that my confusion faded quickly. Three months later, my memory and thinking feel completely back to normal, just as they were before the surgery. I am deeply grateful to the surgical and anesthesia teams for not only removing my tumor but also protecting my mind.

## Data Availability

The original contributions presented in the study are included in the article/Supplementary material, further inquiries can be directed to the corresponding authors.

## Supplementary material

SUPPLEMENTARY MATERIAL 1 CARE Checklist (2013) for this case report.
