# Supplementary material for: Intraoperative cerebral desaturation during low-central-venous-pressure hepatectomy with intermittent Pringle maneuver: a case report
Source: Front Med (Lausanne). 2026 Jun 22;13:1880040. doi: 10.3389/fmed.2026.1880040 (PMC13333627; doi:10.3389/fmed.2026.1880040)
Supplement: SUPPLEMENTARY MATERIAL 1 — CARE Checklist (2013) for this case report. [file Data_Sheet_1.DOCX]

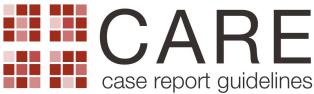
**CARE Checklist (2013) of information to include when writing a case report
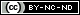
**

**Topic Item Checklist item description Reported on Page**

**Title 1** The words “case report” should be in the title along with the area of focus 1

**Key Words 2** 2 to 5 key words that identify areas covered in this case report 3

**Abstract 3a** Introduction—What is unique about this case? What does it add to the medical literature? 2

**3b** The main symptoms of the patient and the important clinical findings 2

**3c** The main diagnoses, therapeutics interventions, and outcomes 2

**3d** Conclusion—What are the main “take-away” lessons from this case? 2-3

**Introduction 4** One or two paragraphs summarizing why this case is unique with references 3-4

**Patient Information 5a** Demographic information and other patient specific information 4-8

**5b** Main concerns and symptoms of the patient 4-8

**5c** Medical, family, and psychosocial history including relevant genetic information (also see timeline). 4-8

**5d** Relevant past interventions and their outcomes 4-8

**Clinical Findings 6** Describe the relevant physical examination (PE) and other significant clinical findings 4

**Timeline 7** Important information from the patient’s history organized as a timeline 5

# Diagnostic Assessment

**Therapeutic Intervention**

**Follow-up and Outcomes**

**8a** Diagnostic methods (such as PE, laboratory testing, imaging, surveys) 4

**8b** Diagnostic challenges (such as access, financial, or cultural) NA

**8c** Diagnostic reasoning including other diagnoses considered 4-8

**8d** Prognostic characteristics (such as staging in oncology) where applicable 6

**9a** Types of intervention (such as pharmacologic, surgical, preventive, self-care) 5

**9b** Administration of intervention (such as dosage, strength, duration) 5

**9c** Changes in intervention (with rationale) 5

**10a** Clinician and patient-assessed outcomes (when appropriate) 6

**10b** Important follow-up diagnostic and other test results 6

**10c** Intervention adherence and tolerability (How was this assessed?) NA

**10d** Adverse and unanticipated events 5-6

**Discussion 11a** Discussion of the strengths and limitations in your approach to this case 13

**11b** Discussion of the relevant medical literature 11-13

**11c** The rationale for conclusions (including assessment of possible causes) 11-13

**11d** The primary “take-away” lessons of this case report 13

**Patient Perspective 12** When appropriate the patient should share their perspective on the treatments they received 7

**Informed Consent 13** Did the patient give informed consent? Please provide if requested . . . . . . . . . . . . . . . . . . . . . . . . . . . . . . . . . . . . . . . **Yes**
